# Illness perceptions, self-care practices, and glycemic control among type 2 diabetes patients in Chiang Mai, Thailand

**DOI:** 10.1186/s13690-022-00888-1

**Published:** 2022-05-07

**Authors:** Elisha Ngetich, Chanapat Pateekhum, Ahmar Hashmi, Iliatha Papachristou Nadal, Kanokporn Pinyopornpanish, Mike English, Orawan Quansri, Nutchanart Wichit, Sanjay Kinra, Chaisiri Angkurawaranon

**Affiliations:** 1 Nuffield Department of Surgical Sciences, Medical Sciences Division, University of Oxford, Oxford, UK; 2 Department of Family Medicine, Faculty of Medicine, Chiang Mai University, Chiang Mai, Thailand; 3 Department of Non-Communicable Disease Epidemiology, Faculty of Epidemiology and Population Health, London School of Hygiene and Tropical Medicine, London, UK; 4 Department of Psychological Medicine, Institute of Psychiatry, Psychology & Neuroscience, King’s College London, London, UK; 5 Nuffield Department of Medicine, Medical Sciences Division, University of Oxford, Oxford, UK; 6 ASEAN Institute for Health Development, Mahidol University, Salaya, Nakorn Pathom Thailand; 7 Suratthani Rajabhat University, Surat Thani, Thailand

**Keywords:** T2DM, Self-care, HbA1c, Thailand, Self-management

## Abstract

**Background:**

Diabetes Self-Management Education (DSME) is a fundamental aspect of diabetes care, but no standard program exists in Thailand. Understanding current patterns of illness perceptions (concerns) and self-management practices among patients with diabetes in Thailand is vital to develop culturally tailored DSME programs. This study sought to explore the association between reported self-management practices and diabetes perceptions on glycemic control among patients with type 2 diabetes in Chiang Mai Province, Thailand. Specifically, the study examined whether the association between illness perceptions and diabetes control was mediated by self-management.

**Methods:**

This was a cross-sectional study conducted among type 2 diabetes patients on outpatient care and follow-up in four districts hospitals in Chiang Mai, Thailand. Illness perceptions was measured by the Brief Illness Perceptions Questionnaire (BIPQ). Self-management practices were measured by Summary Diabetes Self-Care activities (SDSCA). For illness perceptions and self-management practices, patients were classified into two groups, high level and low level based on the median values. Univariate and multivariable analyses were done to determine the association between the determinant factors: self-care practices and illness perceptions and the outcome of interest- good glycemic control (HbA1c < 7%).

**Results:**

Of the 200 participants recruited into the study, 180 completed the questionnaire. Only 35% of participants had good glycemic control (HBA1c < 7.0). Both illness perceptions and self-management practices were independently linked to glycemic control. Among illness perceptions, a sense of personal control was strongly associated with good glycemic control (*p* = 0.01). For self-management, appropriate diet (*p* = 0.03) and medication adherence (*p* = 0.05) were associated with good glycemic control. After adjustments for key baseline characteristics, patients with high levels of illness perceptions were less likely to achieve glycemic control (OR 0.55, 95% CI 0.29 to 1.14, *p* = 0.11) and those with high level of self-management were more likely to achieve glycemic control (OR 2.11, 95% CI 1.04 to 4.30, *p* = 0.04). The effect size for illness perception attenuated when further adjusted for levels of self-management (OR 0.88, 95% CI 0.39 to 1.96, *p* = 0.75) while the effect size for self-management and glycemic control did not materially change (OR 2.30, 95% CI 1.06 to 5.02, *p* = 0.04).

**Conclusion:**

Illness perceptions and self-management practices are associated with glycemic control. Future culturally tailored interventions in Thailand aimed at improving glycemic should focus on personal control, improving diet and treatment adherence as these are more likely to help improve diabetes control as demonstrated in this study.

## Background

The global burden of diabetes is increasing and is one of the major causes of morbidity and mortality [1, 2]. Thailand has undergone rapid epidemiological, demographic, and nutrition transitions leading to an increasing prevalence of diet-related, non-communicable diseases, such as diabetes [3–5]. The prevalence of type 2 diabetes in Thailand has increased from 2.3% in 1991 to about 8.5% in 2017 and over 4.2 million cases [6]. This growing diet-related non-communicable disease (NCD) burden warrants ways to help improve in the diagnosis, treatment, and management of diabetes.

In diabetes management, great emphasis has been placed on self-management practices—the day-to-day activities patients carry out that promote their health. Diabetic patients are purported to spend only 1% of their time with a healthcare professional. This means that the bulk of management and care for this lifelong disease falls upon patients and their caregivers [7]. Hence, empowering patients with the necessary knowledge and skills to better manage their chronic conditions is key in diabetes care [8].

Significant evidence shows that self-management is effective in improving outcomes such as glycemic control, quality of life, all-cause mortality risk, body mass index and blood pressure [9–14]. Despite having a strong universal health care system and recognizing the importance of self-management for diabetes, Thailand has yet to introduce a national diabetes self-management education program [15]. A study in rural Thailand showed improved self-efficacy and quality of life of a family-oriented, self-management program compared to routine care [16], but it is uncertain if these benefits would persist beyond the short-term [17, 18]. Other Low-and Middle- income countries settings cite cost of continuing education programs, human resource constraints, and logistical challenges as reasons limiting the implementation and long-term benefits of diabetes education programs [19]. These factors also constrain self-management education programs in Thailand [20], for although self-management features prominently in the Thai diabetes management guidelines [21], there are no mechanisms or structures through which healthcare providers can promote self-management for their patients [20].

In Thailand, behavioral change, and self-management education programs for people with diabetes are often offered in outpatient settings. Current efforts are underway to design and evaluate a scalable diabetes self-management education (DSME) program for primary care in Thailand [22, 23]. Not only will programmatic issues need to be addressed, but a successful, widespread diabetes self-management program in Thailand will also need to be guided by behavioral theories and tailored to the local context [24]. Behavioral theory can inform an approach to understanding how perceptions among Thai patients with diabetes influence their behaviors and potential adherence to DSME programs. Leventhal’s self-regulatory model [25]—a well-researched approach—considers an individuals’ cognitive and emotional perceptions as they relate to illness in three stages: (1) forming a representation of the illness; (2) adopting coping behaviors, and (3) appraising the efficacy of these behaviors [25, 26]. Thus suggesting that a patient’s illness perceptions can directly relate to their self-management behaviors [27, 28]. While there is some evidence to suggest that aspects of illness perceptions are associated with medication adherence and attendance rate among patients with diabetes in Thailand [29, 30], very little is known about the illness perceptions and current self-care practices and among diabetic patients in Thailand. Moreover, illness perceptions and self-care practices can also vary between different settings [31, 32].

A better understanding of illness perceptions and their relation to appropriate diabetes self-management practices will provide a stronger, conceptual grounding for development of diabetes self-management education programs in Thailand. This study, therefore, sought to explore the association between reported self-management practices and diabetes perceptions on glycemic control among patients with type 2 diabetes in Chiang Mai Province, Thailand. Specifically, the study examined whether the association between illness perceptions and diabetes control was mediated by self-management.

## Methods

### Study setting, recruitment, and design

This quantitative cross-sectional study was conducted among type 2 diabetes patients in four districts hospitals within Chiang Mai province, Thailand between March, and August 2019. Study participants were selected from those diagnosed with diabetes and on out-patient care and follow-up at any of these four district hospitals. Nurses helped sequentially identify eligible patients and consent was obtained by researcher assistants who had no role in management of the patients. Eligible patients from each site were identified until target recruitment, 50 participants from each site, was reached. Only participants with hemoglobin A1C (HbA1c) tests done in the 6 months prior to the survey were recruited. This was to ensure that glycemic control represented by this HBA1c was reflective of current self-management practices being assessed. Patients that were < 18 years old and those severely ill or cognitively impaired were excluded.

### Data collection, tools and definitions

Self-administered questionnaires were used for data collection with the help of clinical research assistants who were trained before data collection started. Data collected included socio-demographic characteristics (age, gender, occupation, religion), place of routine follow-up, education level, health insurance scheme, average income, illness perception and self-management practices. Clinical data included height and weight, duration with diabetes, insulin therapy and presence of comorbidities.

### Illness perceptions

The Brief-illness perceptions Questionnaire (BIPQ) is a validated tool used to assess illness perceptions among patients with chronic conditions such as diabetes [26]. It has been shown to have good psychometric properties including validity and reliability in over 36 countries [33], including Thailand. The Thai version has been used to explore perceptions among people with hypertension and demonstrated good test–retest reliabilities between 0.75 to 0.97 for each of the eight domains of illness perceptions [34]. The original term “illnesses” used in the questionnaire was replaced with “diabetes” for the purposes of this study. The eight domains of illness perception and questions used to assess were:

1. Consequences: How much does your diabetes affect your life?
2. Timeline: How long do you think your diabetes will continue?
3. Personal control: How much control do you feel you have over your diabetes?
4. Treatment control: How much do you think your treatment can help your diabetes?
5. Identity: How much do you experience symptoms from your diabetes?
6. Concern: How concerned are you about your diabetes?
7. Coherence: How well do you feel you understand your diabetes?
8. Emotional: How much does your diabetes affect you emotionally?

Each BIPQ domain is scored from 0–10, ranging from 0 meaning ‘not at all’ and 10 representing extreme effects upon an individuals’ life, and a spectrum of responses in between. Scores from all 8 questions were summed up after reversing for item 3, 4, and 7 to give an overall score ranging from 0 to 80. BIPQ tool primarily measures negative illness perceptions as indicated by the questions. However, question 3,4 and 7 assess positive illness perceptions hence the need for reversal of scores in these questions in the cumulative score. A high score shows that the participant feels threatened by their diabetes condition. The level of illness for this study was classified into two groups with “high” and “low” illness perception falling above or below the median, respectively. A “high” illness perception indicates a high level of negative perceptions which suggest that the patient may not be coping well with diabetes. Conversely, a “low” illness perception indicates low negative perceptions which suggests that the patients may be coping well with diabetes.

### Diabetes self-management

The Summary Diabetes Self-Care Activities Questionnaire SDSCA [35] was specifically developed to provide more robust measures of self-care practices in a codified manner and has been translated into Thai [36]. The tool is broken down into fifteen questions covering five major aspects of self-management: diet (5 questions), physical activity (2 questions), blood sugar testing (2 questions), medication use (1 question), and foot care (5 questions). Participants were asked how many days in the past seven days did they engaged in appropriate self-care activities related to each of the five major aspects. The overall score was obtained by adding the mean scores for diet, physical activity, blood glucose testing, foot care and medication resulting in a range of scores between 0(lowest) and 35(highest). Participants were categorized as “high” meaning good self-care practices or “low” meaning poor self-care practices using the median cut-off.

### Diabetes control

The latest HBA1C of the participants was obtained from participants’ medical history. HBA1C was considered as a continuous variable and as a categorical variable with < 7% considered good control and poor control (≥ 7.0%) [37, 38].

### Data analysis

Descriptive statistics were summarized as means and standard deviation (SD) for normally distributed continuous variables or median and interquartile ranges (IQR) for variables with non-normal distributions [39]. Univariate analysis was done for all the domains of self-management practices and illness perception with glycemic control using chi-square, t-test or Wilcoxon Rank Sum test as appropriate.

As outlined in the introduction, based on a patient’s illness perception they might adopt appropriate coping strategies and behaviors which should then lead to better glycemic control. A mediation analysis was performed using an approach described by Baron and Kenny [40, 41] to examine whether the association between illness perception and glycemic control was mediated by appropriate self-management practices in the population. We examined the following steps as part of the mediation analysis:

1. Whether there’s an association between illness perception self and glycemic control
2. Whether there’s an association between self-management and glycemic control
3. Whether there’s an association between illness perception and self-management practices
4. Whether the association between illness perception and glycemic control attenuated when self-management (the mediator) was included in the model
5. Whether the association between self-management and glycemic control remain consistent when illness perception was included in the model (not mediated or not confounded by illness perception)

Final multivariable logistic regression models were adjustment for significant baseline socio-demographic and clinical characteristics in univariate analyses.

## Results

### Socio-demographic and clinical characteristics

Two hundred participants were recruited into the study. Of these 180 completed the questionnaire. The mean age of the participants was 63.1(sd 9.0) years, two-thirds (67.1%) of whom were female. Only 35% of the participants had good glycemic control (HBA1c < 7.0%), 40% of participants had BMI higher than 25 which is categorized as obese according to the Asian Pacific BMI chart [42]. The median duration with diabetes was 10.3 (sd 7.7) years. Majority of the participants had primary school level of education (76.7%). Of these baseline and clinical characteristics, gender (female) (*p* = 0.03) advancing age (*p* < 0.01), longer duration with diabetes (*p* = 0.05) and being on insulin therapy (*p* < 0.01) showed a statistically significant association with poor glycemic control (Table 1).

**Table 1:** Socio-demographic and clinical characteristics

	Observation	Poor control (row %)	Good control (row %)	*p*-value
Total sample	180	65.0	35.0	
Age group				< 0.01
< 60	49	81.6	18.4	
60–70	95	62.1	37.9	
> 70	36	50.0	50.0	
Sex				0.03
Male	59	54.2	45.8	
Female	121	70.3	29.7	
Highest education				0.80
Primary school	138	64.6	35.5	
Higher than primary school	42	66.7	33.3	
Monthly income (baht)^a^				0.20
< 2,500	44	56.8	43.2	
2,500–10.000	70	62.9	37.1	
> 10,000	66	72.7	27.3	
BMI				0.34
non-obese (BMI < = 25)	108	62.0	38.0	
obese (BMI > 25)	72	69.4	30.6	
Duration with diabetes				0.05
< 5 years	67	52.2	47.8	
5–10 years	47	70.2	29.8	
10–15 years	32	71.9	28.1	
> 15 years	34	76.5	23.5	
On insulin				0.01
No	156	61.5	38.5	
Yes	23	87.5	12.5	
Self care activity				
Mean score (sd)	158	19.4 (3.9)	20.6 (3.4)	0.07
Low level (score < 20)	89	73.0	27.0	0.02
High level (score > = 20)	78	55.1	44.9	
Illness perception				
Mean score	156	31.0 (11.6)	26.7 (11.2)	0.01
low (illness score < 30)	77	55.8	44.2	0.04
high (illness score > = 30)	81	71.6	28.4	

^a^1 US dollar is worth approximately 33 Thai Baht (in March 2022)

### Illness perceptions and glycemic control

Of the individual domains, the highest median score (10/10) was for “timeline”, indicating that most patients believed that their condition was likely to be permanent rather than temporary. Other concerns were related to the consequences of diabetes in their life (“consequences”) with a median score of 5/10 followed by concerns regarding their ability to control their diabetes with a median score of 4/10 (“concern”). The overall mean score for illness perceptions was 29.4 out of 80 (sd 11.6). Higher level of illness perception was statistically significantly associated with poor glycemic control (*p* = 0.03). Those with poor glycemic control had a mean illness score of 31.0 (sd 11.6) while those with good glycemic control had a mean illness score of 26.5 (sd 11.2) Of all illness perception domains, personal control was strongly associated with glycemic control (*p* = 0.01) (Table 2).

**Table 2:** Illness perception and diabetes control

Illness Domain	Score	Total	Poor control	Good control	*p*-value
Personal control	Mean (sd)	3.60 (2.7)	3.97 (2.7)	2.90 (2.5)	0.01
	Median (IQR)	4.0 (1.0 to 5.0)	5 (2.0 to 5.0)	3 (0.0 to 5.0)	0.01
Treatment control	Mean (sd)	2.68 (2.3)	2.69 (2.3)	2.66 (2.3)	0.94
	Median (IQR)	2.0 (0.0 to 5.0)	2.0 (0.0 to 5.0)	2.0 (0.0 to 5.0)	0.99
Coherence (understand)	Mean (sd)	2.44 (2.6)	2.42 (2.7)	2.49 (2.4)	0.86
	Median (IQR)	2.0 (0.0 to 5.0)	2.0 (0.0 to 4.0)	2.0 (0.0 to 5.0)	0.58
Consequences (affect)	Mean (sd)	3.98 (3.7)	4.21 (3.8)	3.56 (3.5)	0.27
	Median (IQR)	5.0 (0.0 to 7.0)	5.0 (0.0 to 8.0)	4.0 (0.0 to 5.0)	0.37
Timeline (continue)	Mean (sd)	8.68 (2.8)	8.90 (2.6)	8.28 (3.1)	0.16
	Median (IQR)	10 (10.0 to 10.0)	10 (10 to 10.0)	10 (5.0 to 10.0)	0.15
Identity (symptoms)	Mean (sd)	2.70 (3.4)	2.88 (3.6)	2.33 (3.1)	0.31
	Median (IQR)	0 (0.0 to 5.0)	0 (0.0 to 5.0)	0 (0.0 to 5.0)	0.42
Concern	Mean (sd)	2.58 (3.4)	2.82 (3.4)	2.11 (3.3)	0.18
	Median (IQR)	0 (0.0 to 5.0)	0 (0.0 to 5.0)	0 (0.0 to 5.0)	0.15
Emotional	Mean (sd)	2.50 (3.3)	2.72 (3.4)	2.08 (3.1)	0.21
	Median (IQR)	0 (0.0 to 5.0)	0 (0.0 to 5.0)	0 (0.0 to 5.0)	0.18
Total illness score	Mean (sd)	29.4 (11.6)	31.0 (11.6)	26.7 (11.2)	0.03
	Median (IQR)	30.0 (21.0 to 38.0)	31.0 (22.0 to 39.0)	24.0 (20 to 35.0)	0.04

*Sd* Standard deviation, *IQR* Inter-quartile range

^a^ttest for comparisons of means, Wilcoxon rank sum test for comparisons of median

### Self-management practices and glycemic control

Some aspects of self-management, specifically, foot care and medication adherence were practiced daily. The scores were lower for glucose monitoring, diet and physical activity with median scores of 0 (IQR 0 to 0), 2.8 (IQR 0 to 7) and 3.0 (IQR 1.4 to 4.2) respectively. Overall, higher levels of self-management practices were associated with good glycemic control (*p* = 0.04). Among the specific aspects of self-management, increasing self-care in terms of diet (*p* = 0.03) and medication adherence (*p* = 0.05) were significantly associated with good glycemic control (Table 3).

**Table 3:** Self-care activities and diabetes control

SDSCA	Days per week with appropriate self-management	Total	Poor control	Good control	*p*-value*
Diet score	Mean (sd)	3.07 (1.6)	2.88 (1.6)	3.43 (1.6)	0.03
	Median (IQR)	2.8 (1.4 to 4.2)	2.4 (1.4 to 3.8)	3.1 (2.3 to 4.4)	0.02
Physical activity	Mean (sd)	3.24 (2.8)	3.11 (2.9)	3.48 (2.7)	0.41
	Median (IQR)	3.0 (0 to 7)	2.5 (0 to 7)	3.5 (1 to 7)	0.35
Glucose Testing	Mean (sd)	0.22 (0.7)	0.22 (0.8)	0.22 (0.7)	0.93
	Median (IQR)	0 (0 to 0)	0 (0 to 0)	0 (0 to 0)	0.93
Foot care	Mean (sd)	6.48 (0.9)	6.49 (1.0)	6.46 (0.7)	0.52
	Median (IQR)	7 (6 to 7)	7 (6.4 to 7)	7 (5.6 to 7)	0.52
Medication	Mean (sd)	6.79 (0.8)	6.71 (1.0)	6.95 (0.2)	0.05
	Median (IQR)	7 (7 to 7)	7 (7 to 7)	7 (7 to 7)	0.05
Total SDSCA score	Mean (sd)	19.8 (3.8)	19.4 (3.9)	20.6 (3.4)	0.06
	Median (IQR)	19.7 (16.9 to 22.4)	18.8 (16.0 to 22.4)	20.6 (18.5 to 23.4)	0.04

*Sd* Standard deviation, *IQR* Inter-quartile range

^*^t-test for comparisons of means, Wilcoxon rank sum test for comparisons of median

### Multivariable analyses and mediation analyses

**Fig. 1:**
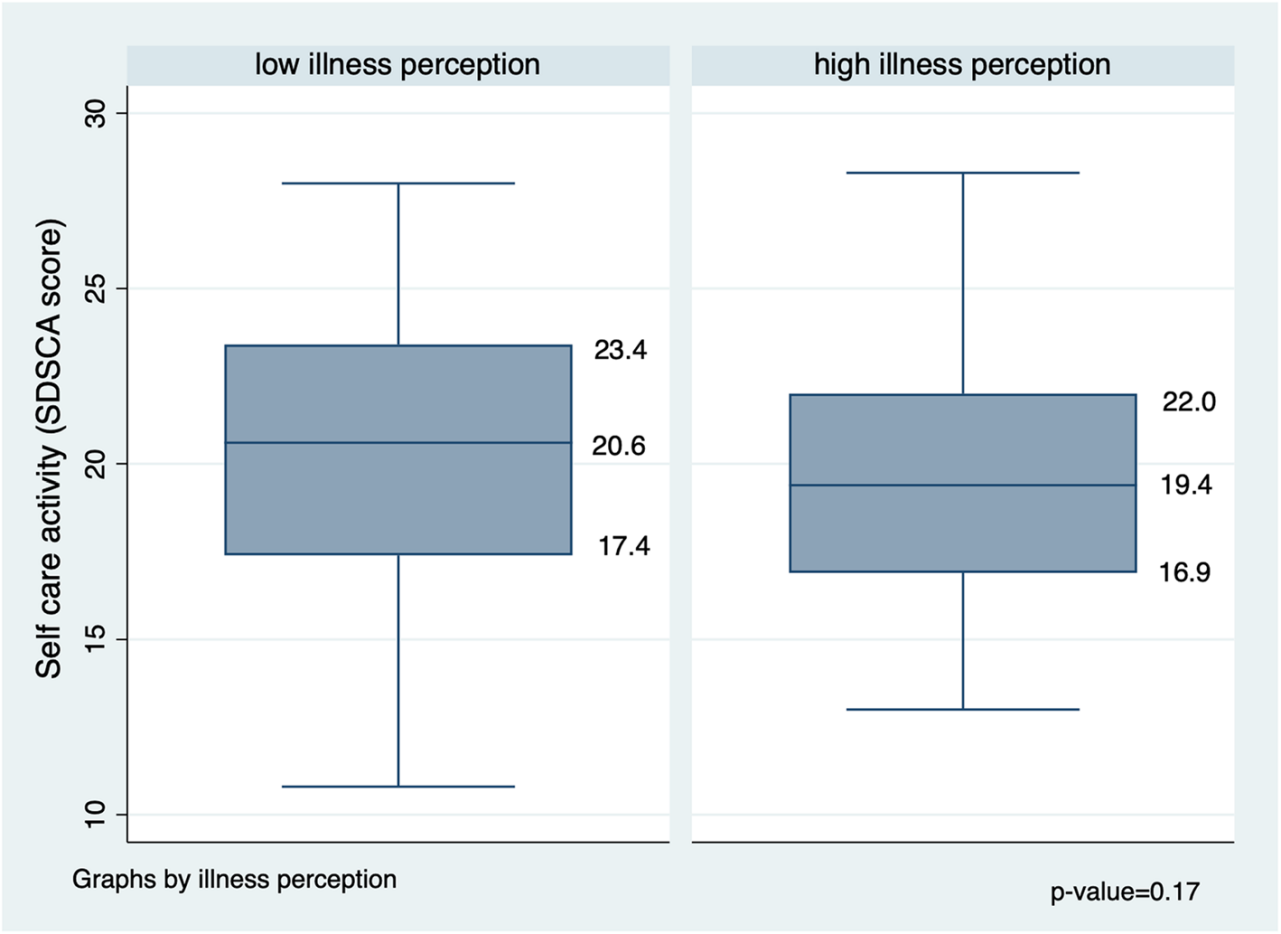
Self-care activity scores by levels of illness perception

Participants with low levels of illness perception had slightly higher levels of self-management (median 20.6, IQR 17.4 to 23.4) compared to those with high levels of illness perception (median 19.4, IQR 16.0 to 22.0) but this did not achieve statistical significance (Fig. 1).

After adjustments for key baseline characteristics, there was some weak evidence to suggest that illness perception was associated with glycemic control. Those with high levels of illness perceptions were less likely to achieve glycemic control (OR 0.55, 95% CI 0.29 to 1.14, *p* = 0.11). The association between self-management and glycemic control was stronger. Those with a high level of self-management were more likely to achieve glycemic control (OR 2.11, 95% CI 1.04 to 4.30, *p* = 0.04) (Table 4 Model 1).

**Table 4:** Self-care activity, illness perception and diabetes control

	Model 1 OR for DM control (95% CI, *p*-value)	Model 2 OR for DM control (95% CI, *p*-value)
Illness perception		
Low	Reference	Reference
High	0.55 (0.29 to 1.14, *p* = 0.11)	0.88 (0.39 to 1.96, *p* = 0.75)
SDSCA		
low	Reference	Reference
High	2.11 (1.04 to 4.30, *p* = 0.04)	2.30 (1.06 to 5.02, *p* = 0.04)

**Model 1**: each exposure (sdsca and Ilness perception) modelled individually and adjusted for age, sex, bmi, location of treatment, duration with diabetes, insulin, and income

**Model 2:** each exposure (sdsca and illness perception) mutually adjusted for each exposure and adjusted for age, sex, bmi, location of treatment, duration with diabetes, insulin, and income

For the mediation analyses, the effect size for illness perception attenuated when further adjusted for levels of self-management practices (OR 0.88, 95% CI 0.39 to 1.96, *p* = 0.75) while the effect size for self-management and glycemic control did not materially change (OR 2.30, 95% CI 1.06 to 5.02, *p* = 0.04) (Table 4 Model 2).

## Discussion

This study explored the association between illness perceptions, self-management practices, and glycemic control among patients with type 2 diabetes in northern Thailand. The most concerning illness perceptions among patients were the aspects related to timeline (diabetes likely to be permanent rather than temporary), consequences (diabetes is affecting my life), and personal control (no control over my diabetes). Higher illness perception scores were associated with poor glycemic control. Low self-management practices, particularly for diet and glucose testing, were also associated with poor glycemic control. The study also demonstrated that the association between illness perception was mediated by self-management practices.

In the current study, just over a third of the participants met the recommended HBA1c cut-off of 7.0%. It is consistent with findings in other studies, where more than 60% of patients often do not hit the recommended glycemic target [43]. This study suggests that higher patients’ illness perceptions about diabetes was associated with poorer glycemic control. One domain in particular, personal control—belief in one’s own ability to control their diabetes—was strongly linked with glycemic control. This finding is supported by previous studies where personal control was the strongest predictor of glycemic control [44, 45], including in the original studies validating the use of the BIPQ [26]. However, in contrast to what was reported in the Broadbent et al. study [26], we did not find an association between glycemic control and the treatment control or identity domains, potentially due to the differences in the study populations. In the study by Broadbent et al., conducted in New Zealand, the mean treatment control illness score was 8.0 while it our study, it was only 2.68. The mean identity illness score was also higher in the Broadbent study compared to our study (4.6 vs 2.7). This reflects that many cultural and socioenvironmental aspects may affect the patient’s illness perceptions, which may include level of education and literacy among the population and how health care is organized and delivered [46]. This finding helps to narrow down the illness perception domains that require specific focus and emphasis in addressing among patients with T2DM in Thailand.

For self-management practices, it is noteworthy that glucose monitoring was not routinely done as this is not covered in the universal health coverage scheme and would therefore be an additional cost to the patient [15]. While this study found good treatment adherence and footcare, there was poor adherence to healthy diet and physical activity. The current study corroborates findings elsewhere in Thailand where only 31% of participants adhered to appropriate diet and exercise regimens [47]. Our current study noted that diet and adherence to medications were the self-management practices most strongly associated with glycemic control and adds to a growing literature that suggests that sub-optimal individual level self-management and structural health systems challenges persist in Thailand [15].

In theory, patients who were less threatened by diabetes (illness perception) would be expected to perform better self-management activities, and consequently, have improved glycemic control [29, 41, 44]. While the results in this study generally supported this statement, there was insufficient evidence for an association between illness perceptions and self-management practices. However, it was difficult to discern the overall framework as the study also demonstrated some evidence that the relationship between illness perception and glycemic control was mediated through self-management practices.

Another important finding from this study is the significant association between patients characteristics including age, gender, duration with diabetes, and glycemic control. Several previous studies have observed that glycemic control is worse in females than age-matched males [48–50]. We also demonstrated that glycemic control is worse in younger patients < 60yrs compared to older patients. These findings have been demonstrated in other studies [51].Our study also showed that glycemic control worsens with increasing number of years with diabetes which has been shown in other studies [52]. These reflect potential risk groups where illness perceptions should be explored.

This current study demonstrates the significant role of self-care practices in influencing diabetes outcomes and the need to focus on changing patients’ perceptions about their illnesses. These findings give credence to current efforts to roll out a structured education program tailored to the Thai population with the aim of empowering patients to take charge of their illnesses. This may help improve diabetes control in Thailand which has stagnated within the 33–36% range from 2012–2018 [53, 54].

The findings of this current study should be interpreted carefully in light of some limitations. First, the questionnaire for assessing self-management practices only collects data about the past seven days and assumes that this is representative of the patient’s daily practice. Although a limitation, the reliability and validity of the SDSCA has been demonstrated in published literature and in other developing Asian countries. In addition, we acknowledge that level of education may lead to more awareness about these self-care behaviours and could result in recall bias. However, the level of education did not significantly differ between those with good control and poor control thus unlikely to cause differential misclassification in reporting of self-care activities. Most of our patients were between 60 and 70 years old and we excluded those with severely impaired cognition, thus our results may not be generalizable to the full spectrum of patients living with diabetes. We used Leventhal’s self-regulatory model as the basis for our analyses. However, there are other behavioral theories, such as social cognitive theory or the theory of planned behavior [55] that could also explain some of the pathways between how illness perceptions, self-management behaviors and glycemic control. However, data on motivation, beliefs, and intention were not available to explore these theories in detailed [56].

## Conclusions

The study describe key illness concerns and area of self-management which could be improved among diabetes patients on outpatient care and follow-up in Thailand. It also provides evidence to support the need to address these illness concerns as a way to promote self-management practices. Particular emphasis should be placed on personal control, improving patient diet, physical activity and treatment adherence. Diabetes self-management programs in Thailand may consider such information in future to help in the development of culturally tailored interventions.

## Data Availability

The dataset used and/or analysed during the current study are available from the corresponding author on reasonable request.

## Abbreviations

BIPQ: Brief illness perceptions questionnaire
DSME: Diabetes Self-Management Education
HbA1c: Hemoglobin A1C
IQR: Interquartile range
NCD: Non-communicable disease
SD: Standard deviation
SDSCA: The summary diabetes self-care activities questionnaire
